# Evidence for Cognitive Placebo and Nocebo Effects in Healthy Individuals

**DOI:** 10.1038/s41598-018-35124-w

**Published:** 2018-11-28

**Authors:** Zsolt Turi, Espen Bjørkedal, Luisa Gunkel, Andrea Antal, Walter Paulus, Matthias Mittner

**Affiliations:** 1 Department of Clinical Neurophysiology, University Medical Center Goettingen, Goettingen, Germany; 2 Institute for Psychology, University of Tromsø, Tromsø, Norway

## Abstract

Inactive interventions can have significant effects on cognitive performance. Understanding the generation of these cognitive placebo/nocebo effects is crucial for evaluating the cognitive impacts of interventional methods, such as non-invasive brain stimulation (NIBS). We report both cognitive placebo and nocebo effects on reward-based learning performance induced using an active sham NIBS protocol, verbal suggestions and conditioning in 80 healthy participants. Whereas our placebo manipulation increased both expected and perceived cognitive performance, nocebo had a detrimental effect on both. Model-based analysis suggests manipulation-specific strategic adjustments in learning-rates: Participants in the placebo group showed stronger learning from losses and reduced behavioral noise, participants in the nocebo group showed stronger learning from gains and increased behavioral noise. We conclude that experimentally induced expectancy can impact cognitive functions of healthy adult participants. This has important implications for the use of double-blind study designs that can effectively maintain blinding in NIBS studies.

## Introduction

Placebos and nocebos are physiologically inert substances or simulated interventions, which produce complex psychobiological responses despite the fact that they do not have any direct therapeutic effects^1–3^. While the term “placebo” describes interventions that produce improvements on the chosen outcome measures, “nocebo” is often used for the opposite^4^. Recent frameworks proposed that the generation and regulation of placebo and nocebo effects can be understood from the perspective of information processing and learning mechanisms^2,3^. According to this view, the treatment context, which surrounds the administration of both active, placebo or nocebo interventions, is actively perceived and interpreted by the individual and can induce placebo and nocebo effects via conscious (e.g., memories, expectations) and non-conscious (e.g., conditioning, emotions) cognitive or affective processes^3,5^.

In the long history of placebo/nocebo research, the fundamental demonstrations of placebo/nocebo effects have been largely confined to the pain or motor domains^6–9^. But are cognitive functions similarly amenable to placebo and nocebo effects? Some studies suggest that although placebo/nocebo treatment can influence the subjectively reported outcome measures (e.g., perceived performance), they do not affect the cognitive performance^10–13^. One explanation for the lack of effect may be that the mode of the placebo treatment, such as caffeine placebo or placebo auditory stimulation, does not produce expectancy effects strong enough to induce cognitive placebo/nocebo effects^10,13^. Other studies indicate that several cognitive functions, such as verbal memory, implicit learning, reward learning and attention, can be influenced by placebo/nocebo interventions^14–18^. However, the mechanisms of how cognitive placebo and nocebo effects are generated, which cognitive functions they can affect and the reason when they do not produce any cognitive effects, are largely unknown.

To study cognitive placebo/nocebo effects, we propose an experimental framework in which we focus on instrumental learning in conjunction with the administration of sham protocols of non-invasive brain stimulation (NIBS) interventions^18^. NIBS interventions are neuromodulatory techniques that are increasingly used to study motor and cognitive functions in humans^19^. Here, we use transcranial direct current stimulation (tDCS), which is the most frequently employed research tool in studies that use electrical current stimulation^20^. With this technique, low-intensity constant currents are passed between two or more electrodes attached to the subject’s scalp. Depending on the duration, intensity and polarity of tDCS and the cortical folding pattern of the individual, the externally applied current during active tDCS can increase or decrease the spontaneous net firing rate of the stimulated brain regions by depolarizing or hyperpolarizing resting membrane potentials^19^. To distinguish the actual physiological effects of this stimulation from any effects related to the stimulation context (such as placebo/nocebo effects), active sham protocols are often used. Active placebo is distinguished from inert placebo by the induction of minor adverse-effects^21^. Active sham is necessary as tDCS is usually associated with noticable adverse effects. At lower stimulation intensities (e.g., 1 mA), those adverse-effects are mild to moderate amounts of cutaneous sensations (e.g., tingling or itching)^22,23^ and proper sham protocols induce similar such effects whithout producing any changes in cortical excitability^24^.

The sham protocols of NIBS are particularly suited for placebo induction because of several reasons. First, NIBS are medical devices that can induce placebo/nocebo effects^18,25–29^, similar to deep brain stimulation^30^ or substances, such as placebo caffeine^15^. However, unlike caffeine, the cognitive effects of NIBS interventions are unlikely to be known to the participants and hence NIBS interventions do not induce strong preconceived expectations. This is a crucial advantage because the placebo- and nocebo-inducing information provided by experimenter will be more salient for NIBS^15^. Further, both active and inert placebo effects can be studied as active sham NIBS protocols induce minor adverse effects, whereas inert sham does not. Finally, using sham NIBS protocols can help to better evaluate results of NIBS studies that use improper blinding. Although most of the commercially available and certified tDCS devices are equipped with double-blind operation mode, a large number of studies still use a single-blind study design, inadequate blinding or no blinding at all^31^. In these studies, the impact of intentional and unconscious preferences, bias mechanisms and expectations from the side of the participants as well as the researchers can eventually lead to an overestimation of its effectiveness^32–34^. Therefore, understanding how these biasing mechanisms are generated and maintained is essential to further understand how the effects of NIBS interventions are induced and to judge the magnitude of the bias inherent in a large body of research.

We investigate the effect of placebo and nocebo interventions on instrumental learning^35^, which is a well-suited behavioral paradigm for studying cognitive placebo and nocebo effects because of two reasons. First, a prominent conceptual framework suggests that the placebo response can be considered a special case of a reward anticipation process, characterized by a neural overlap between anticipating the putative beneficial effects of the treatment and anticipating rewards^36^. In line with this hypothesis, substantial placebo-induced release of dopamine (DA) was detected both in the nigrostriatal and mesolimbic DA-ergic pathways using positron emission tomography (PET)^37–39^, that are key regions in instrumental learning^40^. We argue that this neural overlap provides us the opportunity to study the behavioral and physiological bases of cognitive placebo/nocebo effects^18^. Second, employing computational reinforcement-learning models of instrumental learning, enables us to better understand and further explain covert learning performance by estimating latent variables that cannot be directly observed^41^.

In our earlier study we showed that sham protocols of NIBS interventions in conjunction with placebo-inducing written instructions enhanced reward learning in healthy individuals and that the strength of this effect was modulated by perceived uncertainty of the placebo^18^. However, the placebo manipulation left the subjectively reported expected and perceived cognitive effects of the placebo interventions unaffected, resulting in the unusual situation of a behavioural placebo effect without any subjectively perceived improvements. The goal of the present study is to investigate whether we can also effectively influence explicit expectancy and whether any observed cognitive placebo-effects would be modulated accordingly. Moreover, we asked whether we can also evoke cognitive nocebo-effects, i.e., a reduction in cognitive performance due to combined instruction and intervention, in healthy participants.

To achieve this goal, we introduced a instruction-congruent conditioning procedure^13^ in addition to the application of inactive NIBS and the placebo-/nocebo-inducing written instructions. We implement conditioning by surreptitiously manipulating the trial-by-trial feedback of a well-characterized, monetary-reward based instrumental learning task in order to increase or decrease the subjective difficulty level of the task. We hypothesize that the conditioning will increase positive expectancy (i.e., improvement in cognitive performance) in the placebo and negative expectancy (i.e., decline in cognitive performance) in the nocebo group, respectively. We also hypothesized that the increased positive expectancy will improve performance in the placebo and the increased negative expectancy will impair performance in the nocebo group. Based on our previous study, we also hypothesize that participants in the placebo group will have improved learning from gains, which will be reflected by higher learning rates^18^. Because earlier studies have linked the nocebo effect to reduced DA-ergic signaling^39^, we expect that participants in the nocebo group will show an impaired reward learning performance and suboptimal learning rate from gains, similar to the behavioral pattern demonstrated by older healthy participants with age-related loss^42,43^.

## Results

**Figure 1:**
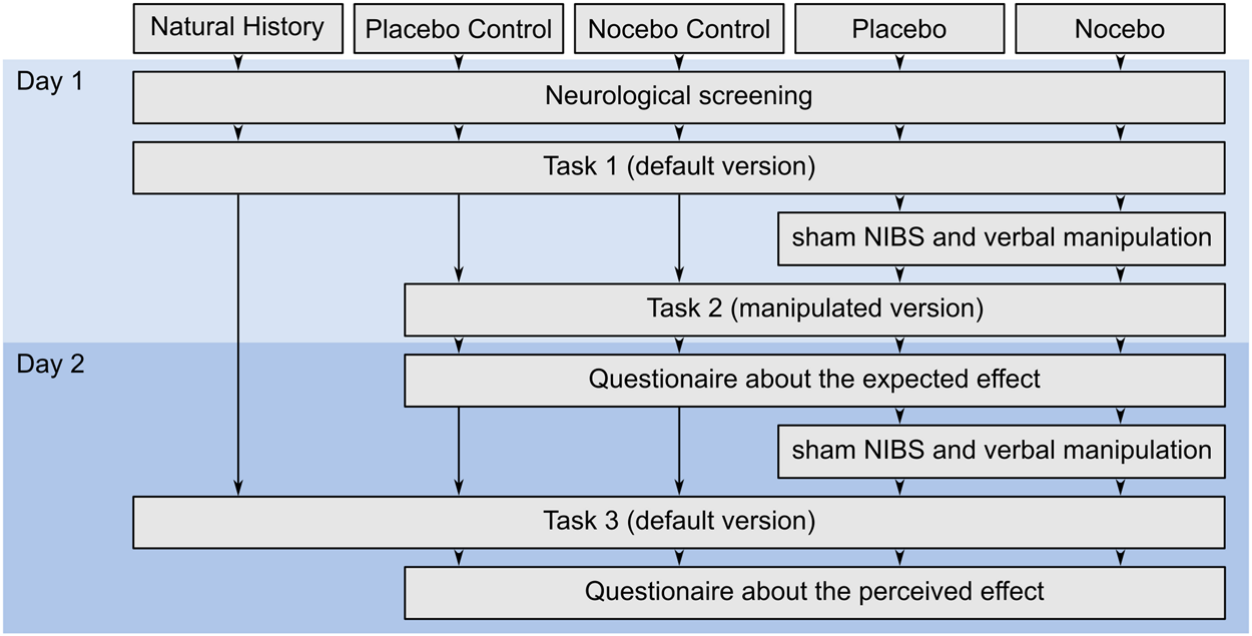
Flow of the participants in the five experimental groups. Abbreviations: NIBS–non-invasive brain stimulation.

The raw data and all reported analyses are available at our repository (https://github.com/ZsoltTuri/2018-placebo-nocebo-study). Participants performed a standard monetary reward-based instrumental learning task^41^. In this task, the participants are repeatedly presented with one of three pairs of Chinese symbols. Each symbol is probabilistically associated with reward and in each pair one symbol has higher reward probability than the other. The goal of the task is to learn to choose the better symbol within each pair and hence accumulate as much reward as possible. In the default version of the task, the reward contingencies of the three symbol pairs were 80/20%, 70/30% and 60/40%. For convenience, we label these pairs as A/B, C/D and E/F pairs. During the experiment, the presentation order of the pairs was randomized. Our study employed a parallel group design, which included five groups: Besides the placebo and nocebo groups, we also collected data from three additional control groups, a placebo control, a nocebo control and a natural history group (NHG). Each volunteer participated in only one experimental group, each of which consisted of 16 participants. All participants came on two consecutive days to complete the two sessions (for an overview see Fig. 1). We used a double-blind study design; neither the participants nor the operator was aware of the genuine purpose of the study.

On the first day, participants in the placebo and nocebo groups performed two tasks. In task 1 (240 trials) default reward contingencies were used in order to assess the baseline performance of the participants. In the shorter task 2 (120 trials), the reward contingencies were manipulated to make the task harder or easier. In addition, the placebo and nocebo groups performed this manipulated version of the task in combination with the sham NIBS application and verbal instructions to its effect. In the placebo group, participants were informed that NIBS would improve their performance and to reinforce these instructions, the reward contingencies were manipulated to be more easily distinguishable (90/10%, 90/10% and 80/20%). In contrast, participants in the nocebo group were provided the information that NIBS would decrease their performance and hence, the reward contingencies were adjusted to make the task 2 more difficult (60/40%, 60/40% and 55/45%). We used the conditioning procedure in task 2, in order to induce instruction-congruent experiences that could potentially increase the expectancy effects in the placebo or nocebo groups on day 2^13^. On the second day, both groups received the placebo or nocebo manipulation again, after which the participants performed task 3 (240 trials). In task 3, we used the default reward contingencies, which allowed us to assess cognitive placebo/nocebo effects. Because the task manipulation could affect the performance independently from the placebo/nocebo treatment, we collected data from two additional control groups. On the first day, the placebo and the nocebo control groups performed task 1 (default version) and task 2 (manipulated version), but without receiving the placebo or nocebo treatment. Participants in the placebo or nocebo control group performed the same manipulated version of task 2 as participants in the placebo or nocebo group, respectively. On the second day, both control groups performed task 3 (default version), without receiving any placebo or nocebo treatment. The two control groups allowed us to characterize the behavioral effects of the task manipulation in task 2 on performance on the second day. We also collected data from a NHG, in which participants received no intervention of any kind and performed task 1 and 3 (both default versions) on two consecutive days.

### Subjective outcome measures

**Figure 2:**
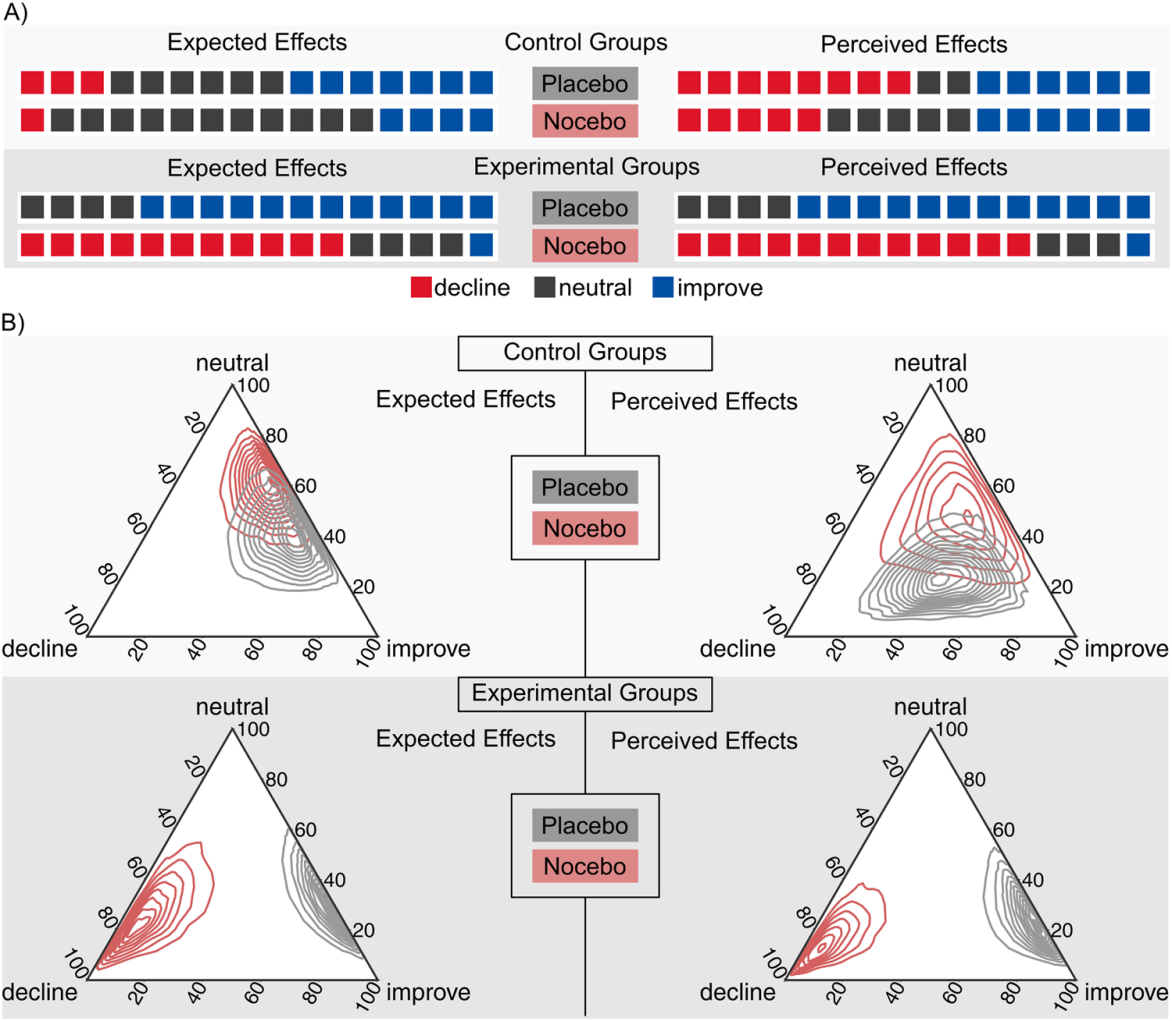
Expected and perceived changes (improve, no change or decline) in the behavioral performance in the four manipulated groups. (**A**) Raw data showing the number of decline, neutral and improve responses per group and question. (**B**) Posterior distribution of the probabilities to respond with one of the three alternatives derived from the softmax-regression model. While the control groups were quite similar in their expectations and in how they experienced their performance in the task, the experimental groups were clearly convinced that the placebo/nocebo manipulation helped/impaired their performance.

Before starting the task on the second day of the experiment, the four manipulated groups were asked to indicate how they expected to perform in the task. They were offered three alternatives: They could either answer that they expected to have a decline or an improvement in performance, or they could indicate that they did not expect any change in their performance (“neutral”). Similarly, after the subjects had completed the experiment on the second day, they were asked whether they experienced any decline or improvement in the task.The data from these two questions were analysed in a Bayesian softmax-regression model^44^ with factors task-manipulation (placebo vs. nocebo), question (expected vs. experienced changes in performance) and group (control vs. experimental groups) including the group × question and group × task-manipulation interactions. The raw coefficients of this model are hard to interpret because they are relative to the reference category and the combination of baseline levels of all factors. To ease interpretation, we therefore calculated the distribution of the posterior probabilities to respond with each of the three response-possibilities (decline, neutral, improve) that is implied by the regression model. Thus, for each group and question, we report probabilities that a member of this group would reply with “decline”, “neutral” or “improve”. The raw data and the results of the softmax-regression are presented in Fig. 2A,B, respectively.

In the placebo group, subjects mainly expected to “improve” (*p*_improve_ = 0.69 [0.49, 0.89]) while expectations for “no change” (*p*_neutral_ = 0.29 [0.09, 0.48]) or a “decline” (*p*_decline_ = 0.01 [0.0005, 0.06]) were much lower. The same pattern of results was observed for the question about experienced performance changes after completing the task (*p*_improve_ = 0.75 [0.54, 0.92], *p*_neutral_ = 0.22 [0.06, 0.42], *p*_decline_ = 0.02 [0.00004, 0.08]). Conversely, participants in the nocebo group expected to “decline” (*p*_decline_ = 0.71 [0.43, 0.99]) while expectations for “no change” (*p*_neutral_ = 0.22 [0.01, 0.46]) or for an “improvement” (*p*_improve_ = 0.07 [0.0009, 0.18]) were much lower. We observed a similar pattern of results for the perceived cognitive performance. Subjects in the nocebo group experienced mostly a “decline” in their performance (*p*_decline_ = 0.81 [0.58, 1.0]) while the probabilities to respond with “improve” (*p*_improve_ = 0.06 [0.00002, 0.16]) or “no change” (*p*_neutral_ = 0.13 [0.0004, 0.30]) were very low. These results are shown in Fig. 2B (lower panel).

In the placebo control group, most subjects reported to expect “improvement” (*p*_improve_ = 0.51 [0.29, 0.73]) or “no change” (*p*_neutral_ = 0.40 [0.20, 0.61]), while the probability for “decline” was much lower (*p*_decline_ = 0.10 [0.001, 0.25]). Participants in the nocebo control group expected mostly “no change” (*p*_neutral_ = 0.61 [0.38, 0.80]) or “improvement” (*p*_improve_ = 0.31 [0.11, 0.51]), while expectations for “decline” were unlikely (*p*_decline_ = 0.07 [0.003, 0.19]). For the perceived performance, participants in the placebo control group reported more likely “improvement” (*p*_improve_ = 0.45 [0.20, 0.70]) and there was a comparable probability to respond with “decline” (*p*_decline_ = 0.32 [0.05, 0.60]) or “no change” (*p*_neutral_ = 0.23 [0.07, 0.41]). In the nocebo control group, participants were more likely to perceive “no change” (*p*_neutral_ = 0.49 [0.19, 0.79]) or “improvement” (*p*_improve_ = 0.37 [0.09, 0.67]) and less likely to perceive “decline” in their performance (*p*_decline_ = 0.14 [0.007, 0.40]). These results are shown in Fig. 2B (upper panel). Thus, the pattern of results in the two control groups was very different from the corresponding placebo/nocebo groups. While the placebo control group had much lower expectations for improvement relative to the placebo group, the nocebo control group were unlikely to expect and perceive decline in their cognitive performance. These results indicate that the placebo and nocebo interventions had a strong effect on subjectively expected and experienced performance changes.

In summary, the results confirmed that the placebo/nocebo manipulation affected both the subjectively-reported expected and perceived cognitive performance that went beyond the effect of the task-manipulation alone. While participants in the placebo group predominantly reported both expected and behavioral improvement, participants in the nocebo group more frequently reported to expect and experience behavioral decline.

### Behavioral Performance

To investigate how performance was affected by the placebo/nocebo manipulations, we performed a model-selection procedure in which we added a factor coding for group to a base model. We used a parallel strategy for reaction times and accuracy and fit Bayesian hiearchical regression models to the data. For the accuracy outcome, we used a logistic regression model. We define reaction time as the time (measured in milliseconds) between the presentation of the symbol pairs and the response of the participant. Accuracy was defined as the proportion of responses to the symbol with higher reward probability (symbols A, C and E). The base model for both accuracy and reaction time data included a main effect of trial to account for the learning effect over time. Further, we added a main effect for symbol-pair to account for the differences in difficulty levels. We also added a trial × symbol pair interaction to capture different learning speeds for the harder and easier symbols. Furthermore the model contained a main effect of day and day × trial interactions. Finally, we added a random intercept for each participant and allowed both trial and symbol pair to vary per participant by adding it as a random slope. The base-model was constructed by successively adding the individual variables to a null model. At each step of the process, the newly added variable improved the model fit in terms of the LOOIC.

Next, we tested two additional models. In the first model, we extended the base model by adding group as main effect, in the second model we also added the group × day interaction that was of most interest theoretically. To decide which model to select, we use the leave-one-out cross-validation information criterion (LOOIC^45^, see Methods for details). The model selection procedure clearly favoured the model that included both group and the group × day interaction for both outcome measures (accuracy: ΔLOOIC = 58.63, RT: ΔLOOIC = 121.63). This provides evidence for the notion that the groups showed differences in performance on the second day after the placebo/nocebo manipulations.

**Figure 3:**
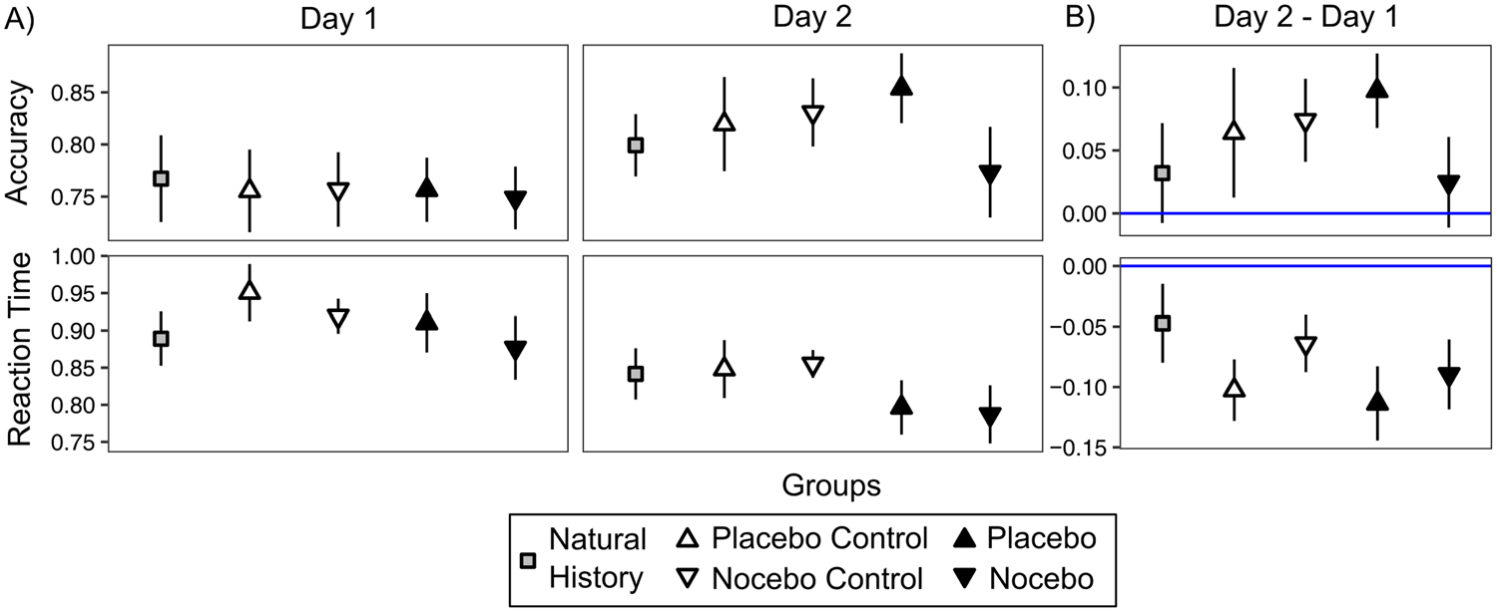
Mean behavioral performance for accuracy (proportion of correct responses) and reaction time (seconds) from day one and day two (**A**) and the difference between day two and day one (**B**). The blue horizontal lines indicate no difference between days, positive values in accuracy indicate improved performance on day two, whereas negative values in reaction time indicate faster respones. Error bars represent standard error of the mean.

Analyzing the model for accuracy further, there was a positive main effect for the trial-number, *b* = 0.71 [0.58, 0.83], indicating learning over the course of the experiment (for a summary of model coefficients, see Table 1). We also observed main effects for symbol pairs CD, *b* = −0.22 [−0.41, −0.02] and EF, *b* = −1.22 [−1.45, −1.00], suggesting that more difficult symbol pairs with less reliably separable reward contingencies were harder to learn than the easier AB pair. The main effect of day, *b* = 0.24 [0.12, 0.36] indicates that participants irrespective of group-membership performed better on the second day (see Fig. 3A). The day × group interaction (see Fig. 3B) revealed a further improvement on day 2 in the placebo group, *b* = 0.55 [0.38, 0.73] relative to the NHG. In contrast, we observed no day × group interaction in the nocebo group, *b* = −0.07 [−0.23, 0.10] indicating no additional decline or improvement compared to the NHG from the first to the second day in this group. However, perhaps surprisingly, also both the placebo control, *b* = 0.24 [0.08, 0.42] and the nocebo control group, *b* = 0.32 [0.15, 0.49] showed an improvement relative to the NHG group. Possibly, this effect is due to the fact that both the placebo control and nocebo control groups were exposed to an additional, albeit manipulated version of the task which might have lead to a slight learning effect over and above that observed in the NHG. We therefore have to compare the effect of the placebo stimulation to their respective control group. The improved learning effect was more pronounced in the placebo group when compared to the two active control groups (placebo-placebo control: *δ* = 0.31 [0.13, 0.49]; placebo-nocebo control: *δ* = 0.23 [0.05, 0.42]) and there was a decline in performance in the nocebo group relative to the two active control groups (nocebo-nocebo control: *δ* = −0.39 [−0.56, −0.21]; nocebo-placebo control: *δ* = −0.31 [−0.47, −0.14]).

**Table 1:** Summary of model coefficients (group-level) for accuracy (logistic regression model) and reaction times (milliseconds).

	Accuracy Mean/95% HDI	Reaction Times Mean/95% HDI
Intercept	2.18 [1.77, 2.59]	876.37 [810.55, 948.43]
Trial (z-score)	0.71 [0.58, 0.83]	−71.92 [−80.23, −63.36]
Pair CD	−0.22 [−0.41, −0.02]	10.73 [−4.05, 26.42]
Pair EF	−1.22 [−1.45, −1.00]	31.98 [16.66, 47.34]
Day	0.24 [0.12, 0.36]	−47.59 [−57.18, −38.27]
Trial × Pair CD	−0.03 [−0.11, 0.05]	0.40 [−4.78, 5.54]
Trial × Pair EF	−0.28 [−0.35, −0.20]	5.48 [0.44, 10.63]
Trial × Day	0.05 [0.00, 0.11]	17.25 [13.16, 21.38]
Group Placebo	−0.10 [−0.56, 0.37]	17.98 [−74.64, 111.93]
Group Nocebo	−0.09 [−0.54, 0.36]	−8.08 [−103.47, 82.07]
Group Placebo control	−0.09 [−0.57, 0.41]	61.61 [−31.98, 154.23]
Group Nocebo control	−0.26 [−0.74, 0.21]	25.76 [−65.83, 116.78]
Day × Group Placebo	0.55 [0.38, 0.73]	−66.76 [−80.52, −53.26]
Day × Group Nocebo	−0.07 [−0.23, 0.10]	−42.85 [−56.56, −29.08]
Day × Group Placebo control	0.24 [0.08, 0.42]	−55.55 [−68.71, −42.20]
Day × Group Nocebo control	0.32 [0.15, 0.49]	−17.31 [−31.07, −3.90]

In the parallel model for the reaction time, there was a main effect for the number of trial, *b* = −71 [−80, −63], which suggests that participants responded faster over the course of the experiment. The main effect for symbol pair CD, *b* = 11 [−4, 26] and EF, *b* = 32 [17, 46] indicates that participants had longer reaction time for the more difficult symbol pairs, though the HDI for the CD-pair effect did not exclude zero. Also, participants responded faster in the second, when compared to the first day, indicated by the main effect of day, *b* = −48 [−57, −38]. The group × day interactions indicate that the RT improvement from day 1 to day 2 was more pronounced in all groups compared to the NHG (placebo: *b* = −67 [−81, −53]; nocebo: *b* = −43 [−57, −29]; placebo control: *b* = −56 [−69, −42]; nocebo control: *b* = −17 [−31, −4]). The reaction time improvement of the placebo group from the first to the second day were higher when compared to both the nocebo (*δ* = −24 [−38, −11]) and the nocebo control group (*δ* = −49, [−62, −36]) but not reliably when compared to the placebo control group (*δ* = −11, [−24, 2]). The amount of reaction time improvement from the first to the second day was higher for the nocebo group compared to the nocebo control group (*δ* = −26, [−38, −1]) but somewhat lower compared to the placebo control group (*δ* = 12, [0, 26]).

In summary, we observed a placebo and a nocebo effect on the accuracy of the decisions, as it was increased and decreased in the placebo and nocebo groups, respectively, when comparing them to their control groups. The results are less clear for the reaction time data where the placebo group responded fastest while the nocebo group did not show a clear effect.

### Modeling

Performance in the task described above is complex to evaluate since it depends on the knowledge about the value of the respective stimuli that is being acquired by random reward sequences for each subject. The regression models reported above can only roughly account for this learning process. We therefore used reinforcement learning models that are specifically tailored to the task used in our study^18,41^. These models can explain individual learning trajectories, taking into account every single choice and reward outcome for each subject and session (see Methods for details). In a nutshell, the result of fitting the models to behavioural data are individual parameter estimates that describe aspects of an individuals’ behaviour. Those parameters are two learning rates, *α*_*G*_ and *α*_*L*_, respectively and a “noise”-parameter *β*.

The learning rates quantify to which degree new information about the value of a response option, the prediction error, are used to update the estimate of each actions’ value. Low values indicate a slow and gradual accumulation of the value estimate while high values (close to one) characterize a strong updating behaviour were participants quickly change their mind about the relative value of the response-options. We included two separate learning-rates, one for learning from gains and one for learning from losses, because there is evidence for distinct neural mechanism underlying both processes^41^. The noise- or exploration-parameter *β* specifies to which degree the decision about which action to take is based on the difference of the two values. Higher values of this parameter indicate a more random style of decision-making, i.e., that the less-valued option has a higher likelihood to be selected. It is important to note that different combinations of parameter values (*α*_*G*_, *α*_*L*_, *β*) are more or less optimal for decision-making. Increases in learning rate, e.g., are not always beneficial and a certain amount of “noise” (or exploration) is necessary for successful decision making.

We fitted hierarchical versions of this model to the data from all individuals, days and trials simultaneously. In such a hierarchical model, group-level estimates of the parameters constrain single-subject estimates to reduce overfitting. In a model-selection framework, we started with a simplified model featuring a single learning rate and noise-parameter for each individual and compared it to a model that allowed parameters to differ between days and groups. This second model was clearly selected (ΔLOOIC = 52.2). When comparing this model to the full model where learning rates were separated for gains and losses and all parameters were allowed to vary by day, the more complex model again clearly outperformed the simpler one (ΔLOOIC = 87.8). Based on the results of this model-selection procedure, we used the last model for all inferences reported here.

**Figure 4:**
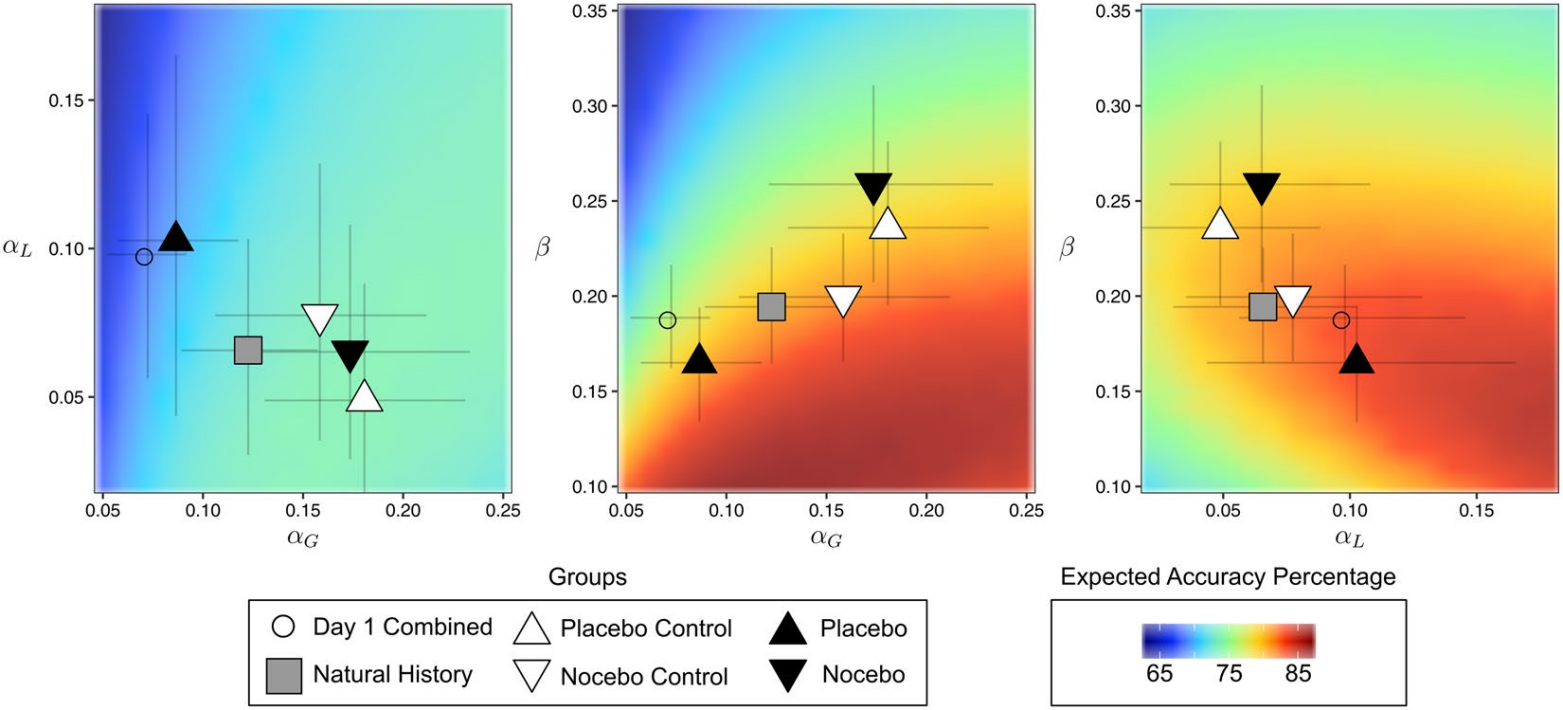
Group-level estimates for the reinforcement-learning model. All three pairs of parameters are plotted, symbols indicate different groups, error-bars are 95% highest-density intervals. Background shows theoretically expected average performance (accuracy) of for each combination of the model-parameters. Red regions indicate a more optimal setting of the parameters, blue regions are less optimal.

The results of this analysis are reported in Fig. 4 and Table 2. Relative to the first day, there was a general increase in learning-rate from gains, ${b}_{{\alpha }_{G}}=0.30$ [0.19,0.40] and reduced learning from losses, ${b}_{{\alpha }_{L}}=-\,0.22$ [−0.14, −0.06]. The exploration parameter *β* was not globally affected, *b*_*β*_ = 0.03 [−0.08, 0.13]. Most importantly, the placebo and nocebo groups showed opposite patterns with respect to all model-parameters: While subjects receiving a placebo manipulation down-regulated their learning-rate from gains, ${b}_{{\alpha }_{G},{\rm{placebo}}}=-\,0.2$ [−0.36, −0.04], it was increased in the nocebo group, ${b}_{{\alpha }_{G},{\rm{nocebo}}}=0.22$ [0.04, 0.41]. In contrast, the placebo manipulation resulted in increased learning from losses, ${b}_{{\alpha }_{L},{\rm{placebo}}}=-\,0.24$ [0.01, 0.48], while the nocebo manipulation did not manifest in changed learning-rates for losses, ${b}_{{\alpha }_{L},{\rm{nocebo}}}=-\,0.01$ [−0.23, 0.21]. Similarly, the group receiving a placebo manipulation showed a reduced tendency to make exploratory decisions, *b*_*β*, placebo_ = −0.16 [−0.31, −0.02] while the nocebo grouped had an increased parameter, *b*_*β*, nocebo_ = 0.28 [0.12, 0.46]. As displayed in Fig. 4 and also reflected in the performance for these two groups, these adjustments resulted in a more optimal parameter setting for the placebo group and a less-optimal parameter setting for the nocebo group when compared to the parameters on the first, unmanipulated, day. These group-specific changes in the reinforcement-learning parameters may reflect an expectation-guided adjustment process: Because participants in the placebo group expected to have an improved performance, they were more surprised by losses (and less surprised by gains) and therefore more strongly integrated information from losses (and less strongly from gains) into their value-estimates. Similarly, participants in the nocebo group who expected to do badly may have preferably opted to learn from the more surprising gains.

**Table 2:** Coefficient estimates for the model-parameters for the five experimental groups.

	Parameter		
	*α*_*G*_	*α*_*L*_	*β*
NHG (baseline)	0.30 [0.19, 0.40]	−0.22 [−0.39,−0.06]	0.03 [−0.08, 0.13]
Placebo	−0.20 [−0.36, −0.04]	0.24 [0.01, 0.48]	−0.16 [−0.31, −0.02]
Nocebo	0.22 [0.04, 0.41]	−0.01 [−0.23, 0.21]	0.28 [0.12, 0.46]
Placebo Control	0.25 [0.09, 0.41]	−0.16 [−0.42, 0.09]	0.19 [0.04, 0.36]
Nocebo Control	0.16 [−0.01, 0.33]	0.08 [−0.15, 0.32]	0.03 [−0.12, 0.17]

Values indicate effects relative to the parameter estimate from day 1 across all groups. For the learning rates *α*_*G*_ and *α*_*L*_, the effects are on the logit-scale, for the noise-parameter *β*, the effect is on the exponential scale (see Methods for details). Values are posterior means and 95% highest-density intervals.

Interestingly, also one of the groups receiving only a task-manipulation but no placebo/nocebo stimulation showed some parameter changes. The group receiving an easier task on the first day (placebo control group) showed a pattern similar to the nocebo group with an increased learning rate from gains, ${b}_{{\alpha }_{G},{\rm{placebocontrol}}}=0.25$ [0.09, 0.41], an unchanged or slightly reduced learning rate from losses, ${b}_{{\alpha }_{L},{\rm{placebocontrol}}}=-\,0.16$ [−0.42, 0.09] and an increased exploration parameter, *b*_*β*, placebocontrol_ = 0.19 [0.04, 0.36]. The nocebo control group who received a harder task on the first day but no instructions behaved in a similar way as the NHG, ${b}_{{\alpha }_{G},{\rm{nocebocontrol}}}=0.16$ [−0.01, 0.33], ${b}_{{\alpha }_{G},{\rm{nocebocontrol}}}=0.08$ [−0.15, 0.32], *b*_*β*, nocebocontrol_ = 0.03 [−0.12, 0.17]. Perhaps subjects in the placebo control group who performed an easier version of the task on day one were surprised by how hard the task on the second day turned out to be and therefore experienced a nocebo-type effect. This interpretation matches with the fact that also the subjectively reported performance decreases in this group were quite similar to those obtained from the nocebo group.

## Discussion

We hypothesized that the administration of a conditioned placebo treatment would induce expectations about improved cognitive functions and lead to an improved performance on an instrumental learning task. We anticipated the opposite effects for the conditioned nocebo treatment. We tested these hypotheses by administering active sham protocols of NIBS in combination with verbal suggestions of enhanced performance (placebo group) or decreased performance (nocebo group) between two identical instrumental learning tasks. Moreover, we reinforced these suggestions by giving participants in these groups a manipulated version of the task without their knowledge. Participants in the placebo group got more trial-by-trial positive feedback, while participants in the nocebo group got more trial-by-trial negative feedback. A natural history group controlled for the effect of repeated testing, while the placebo and nocebo control groups controlled for the effect of task manipulation. This study design allows us to conclude that any improvements in performance in the placebo group and/or decreased performance in the nocebo group were due to the placebo treatment.

Our data indicate that the placebo/nocebo intervention increased both the expected and perceived performance in the placebo and decreased them in the nocebo group compared to the control groups. In addition, both placebo and nocebo effects were manifest in response accuracy which was improved in the placebo and decreased in the nocebo group. Reaction time improvement from the first to the second day in all groups were more pronounced in the placebo group while the nocebo group did not show decreased reaction times. We also observed that both control groups showed increased accuracy relative to the natural history group, which suggests an additional practice effect unspecific to the nature of the task manipulation. Possibly, this additional practice effect is due to the fact that the four experimental groups performed the manipulated version of the task on the first day. However, the additional practice effect was abolished in the nocebo group, who performed at the same level as the natural history group (where participants performed no additional task on the first day) and worse than the other two control groups or the placebo group. This suggests that negative expectations of performance interfered with instrumental learning. Thus, practice increased instrumental learning performance and this effect is further boosted by positive and attenuated by negative expectations.

A model-based analysis using a computational reinforcement-learning model provided further insights into how the expectancy manipulation changed the participants’ learning. While the placebo group down-regulated their learning-rate from gains and increased their learning-rate from losses, the nocebo group showed an opposite pattern where the learning rate from gains was increased. This general pattern may be attributed to the notion that subjects in the different groups considered either gains or losses to be more surprising, depending on whether they were told that they would show improvement (placebo) or decreased performance (nocebo). During the placebo treatment on the first day, participants performed an easier version of the instrumental learning task, in which the reward contingencies were manipulated in order to make them experience more positive feedback (i.e., gains). Therefore, participants would quickly learn the better option and stay with it even after the occasional loss. Because we used the default reward contingencies on the second day, participants were likely to experience more negative feedback (i.e., losses) compared to the manipulated task on the first day. Experiencing more negative feedback could interfere with the generation of placebo response and to compensate for this potential attenuating effect, participants were informed that their performance was improved by the placebo treatment and they would perform a more difficult task this time. Therefore, on the second day participants were probably focussing more on loss trials, leading to the increased learning rate from losses. Despite the purportedly harder task, participants expected better performance on the second day. Thus, gain trials were not surprising for them, which lead to more gradual adjustments in the learning rate.

Conversely, subjects in the nocebo group performed a manipulated task with less separable reward contingencies and therefore encountered more negative feedback. Because on the second day we used the default reward contingencies, participants had a higher chance of experiencing more gains compared to the manipulated task on the first day. These subjects received the information that their performance was decreased by the nocebo treatment and they would perform an easier version of the task. Because of the negative expectations about the cognitive performance, trials in which subjects experienced gains were surprising, resulting in stronger learning rate for gains. In addition to the effects on the learning rate, there were differential effects on the exploration parameter that controls to what extent the subjects’ choices were dictated by the learned value difference of the stimuli. Higher values in this parameter indicate that subjects behaved more randomly (or exploratory) and less according to the information learned from the previous encounters of the stimuli. While a certain extend of exploratory behaviour is beneficial for performance, expected performance drops when choice behaviour is too noisy. In the placebo group, the exploration parameter was reduced relative to the other groups which resulted in a parameter setting for this group that was more optimal – hence resulting in the observed performance increases. Conversely, the same parameter was increased in the nocebo group, which resulted in a less optimal parameter setting.

Together, the differential adjustment to the three model-parameters may indicate that the induced expectations led the subjects to employ different strategies when solving the task. In an earlier study, we found that the placebo treatment led to increased learning rates from gains without affecting learning rates from losses or the exploration variable^18^. At first glance, these results may appear to be in conflict with the present findings. However, a crucial difference between the two studies was the way in which the placebo manipulation affected the subjective expectations. While we were able to induce clear and strong expectations in the current study, there was no such effect in our previous study even though we still observed behavioural cognitive placebo effects. The effects observed in the current study may therefore reflect changes to performance that are due to more conscious adjustments in processing (i.e., model-based processing) while the effects from our previous study may have manifested on a lower level of processing (i.e., the model-free system)^18^.

This distinction between two different control system, a model-free and a model-based system, is well known in computational accounts of human instrumental learning^46,47^: The model-free control system employs reflexive control and is guided by past reinforcement history. It relies on computing the prediction error and underpins automatic, habitual stimulus-response behavior. On the contrary, the model-based control system is under reflective control, which supports the mapping of action-outcome contingencies into a coherent, internalized world model. The model-based control system allows explicit prospective planning of future sequences of actions and can therefore overwrite past reinforcement history. Unfortunately, the task employed in the current study is not appropriate to arbitrate between these two systems: A change in both an explicit cognitive strategy or mere low-level changes to the model-based learning system would both be reflected in an adjustment of the same set of parameters. Future studies could therefore aim to investigate this distinction more directly by employing an instrumental learning task, which can distinguish the relative contribution of the model-free and model-based control systems^48–52^ and precisely determine, which learning mechanisms are affected by the different forms of placebo and nocebo treatment.

In the present study, we showed that active sham protocols of NIBS together with placebo/nocebo inducing written instructions and conditioning can affect the expected and the perceived cognitive performance. However, expectations have at least three components. The first component is its direction; for example whether participants expect decline, no change or improvement in cognitive performance. The second component is the amount of expected change, for example the anticipated change in cognitive performance measured by a Likert-scale. The third component refers to the subjective certainty attached to those expectations. For instance, participants may expect their performance to improve somewhat, but they are unsure whether the treatment actually works. In order to gain further knowledge about the development and maintenance of cognitive placebo/nocebo effects in different treatment characteristics, future work should collect data about all three components of expectations. Previous research show that sensory information is processed differently under certain compared to uncertain expectations^18^. Other studies showed that the placebo effect can be stronger if subjects are deceptively told that they get a treatment compared to when they are told it is a 50% chance of receiving treatment or placebo^53^.

Traditionally, instrumental learning is associated with activity of various neuromodulators including the nigrostriatal and mesolimbic DA-ergic, the dorsal raphe serotonin and the locus coeruleus noradrenergic pathways^54^. Based on the observed pattern of our behavioral findigns, we might expect that the DA-ergic activity in the frontal cortex-basal-ganglia loop is a likely neurobiological mechanism involved in generating cognitive placebo/nocebo effects in instrumental learning. The exact neurobiological mechanisms that lead to the observed pattern of behavioral results in the present study have yet to be identified in future neuroimaging studies.

One limitation of the present study is that the diverging information about task difficulty given to the participants on the second day could have biased their motivation towards focusing more or less on the task, which in turn could have improved or decreased their performance. Whereas the placebo group were informed that they would perform a purportedly harder task on the second day, the nocebo group were informed that they would perform a purportedly easier task. Contrary to the instructional manipulation, all groups performed the same default version of the task. However, this instructional manipulation in the placebo group might have motivated the participants to focus even more on the purportedly harder task, whereas it might have demotivated the participants in the nocebo group, so that they focused less on the purportedly easier task. Note that these groups received this extra instructional manipulation due to the conditioning procedure used on the first day. Whereas the placebo group performed a de facto easier task in task 2 on the first day (in order to boost the cognitive placebo effect), the nocebo group did a de facto harder version of the task (in order to boost the cognitive nocebo effect). Because on the second day both groups did the default version of the task, participants in the placebo group were likely to receive more monetary loss than the day before, whereas the nocebo group were likely to receive more monetary gains. This effect could have compromised the placebo/nocebo effects and we therefore used these additional instructional manipulations in order to minimize these effects. In order to reduce the potential motivational and behavioral influence of these extra instructional manipulations, participants were informed in all groups to receive performance-dependent monetary bonus, similar to^18^. As part of this monetary bonus system, the volunteers could increase their final earnings by 70%. Due to performance-dependent monetary bonus, we motivated participants in all groups to engage in a similar and comparable way in the task.

Our results may be reminiscent to studies investigating the effects of stereotype threat/boost which often find that cognitive performance can change as a function of an activated stereotype in stereotype-relevant tasks (e.g., gender in mathematics)^55,56^. These studies suggested that explicit or even implicit activation of negative/positive stereotypes may elicit affective (e.g., performance expectation, individuation tendencies), cognitive (e.g., negative thinking, mind-wandering) and motivational mechanisms (e.g., dejection, effort) that may in turn hinder or boost cognitive performance^55^. Nevertheless, social stereotypes are formed through the socialization process. Conversely, they are activated rather than induced during the experimental manipulation. In contrast, expectancy induced by the experimental manipulation is on a much shorter time-scale when compared to the social stereotypes. In addition, the direction of the effect of our experimental manipulation is arbitrary (i.e., we can induce either positive or negative performance/expectation effects using the same intervention). So far, it was less known whether and to what extent experimentally induced expectancy can impact cognitive functions of healthy adult participants.

Factors contributing to the generation and to the maintenance of cognitive placebo/nocebo effects are particlarly interesting for interventional methods such as NIBS. In case of tDCS and transcranial alternating current stimulation (tACS), there is a current debate whether low intensity stimulation (e.g., 1–2 mA) generates strong enough intracranial electric field in the neural tissue to induce any neurophysiological effects^57–62^. Despite the large body of promising behavioral and physiological findings, researchers have yet to identify and confirm the specific mechanisms involved in the generation of the presumed effect and aftereffects. We conclude that experimentally induced expectancy can impact cognitive functions of healthy adult participants. Therefore, we have shown that it is crucial that cognitive placebo and nocebo-effects are taken into account when designing and evaluating the results of tDCS and tACS studies. Our present findings emphasize the need for a double-blind study design and the proper maintainance of effective blinding because of the presence of strong expectation effects in NIBS studies.

## Methods

### Participants

Eighty male volunteers participated in the study (mean ± SD age: 24.61 ± 3.53 yrs). Because gonadal steroid level could influence reward information processing, female participants were excluded from the study^63^. Before participation, all volunteers were screened by a neurologist at the Department Clinical Neurophysiology, University Medical Center Goettingen and gave written informed consent. None of the participants had a history or presence of current medical, neurological or psychiatric illnesses including epilepsy, drug and/or alcohol addiction and the presence of metal implants in the head, neck and chest. None of the participants were familiar with Chinese or Japanese characters. The experiment, experimental protocols and all methods used in the present study was approved by the Ethic Committee of the University Medical Center Goettingen and was performed in accordance with relevant guidelines and regulations.

### Operator

The operator, who was responsible for the recruitment and data collection of the placebo and nocebo groups, was a native German, female dentistry student, naїve to tDCS and transcranial near infrared light stimulation (tNILS) studies and had no prior experience with NIBS research.

### Paradigm

Participants performed a probabilistic learning and decision-making task^41^. In this task, participants are repeatedly presented with one of the three symbol pairs (represented by Chinese symbols) and the goal is to learn to choose the better symbol within each pair. Each symbol is probabilistically associated with reward and in each pair one symbol has higher reward probability than the other (e.g., 80/20%, 70/30% and 60/40% for A/B, C/D and E/F). The exact values for the reward contingencies are unknown to the participants.

Each trial started with a fixation cross for 0.3 s, followed by the symbol pair for 1.7 s. Participants were required to make their responses within 1.7 s. In each trial, the reaction time was defined by the time delay in milliseconds between the start of presenting the symbol pair and pressing one of the two response buttons on the designated response pad (RB-740, Cedrus). In order to select the left symbol from the pair, participants were required to press the corresponding button indicated by customized key top with a letter L. For selecting the right symbol on the screen, a customized key top with a letter R was used. Note that the letters L and R correspond to the initials of the words indicating the left (links) or the right (rechts) directions in German language. The L and R keys were located on the left and right side of the response pad, respectively. The presentation side, whether the symbol appears on the left or right side on the screen, was counterbalanced. The symbol selected by the participant was highlighted for 0.5 s, followed by a feedback lasting for 0.5 s.

The first and the third task consist of four experimental blocks, whereas the second consisted of two blocks. In each block, the three symbol pairs were randomly presented 20 times. The presentation of each symbol pair is counterbalanced for the left and right side in each block (i.e., AB or BA). The first and the third task employed baseline reward contingencies (80/20%, 70/30% and 60/40%), whereas the second task utilized manipulated reward contingencies. Since participants in the placebo group were informed that the stimulation would improve their performance, the reward contingencies were more reliably separated in the second task (90/10%, 90/10% and 80/20%). On the contrary, participants in the nocebo group were provided the information that the stimulation would decrease their performance; hence, reward contingencies were less reliably separated (60/40%, 60/40% and 55/45%). The reward contingencies in the second task are based on two internal pilot studies involving nine participants altogether. Based on the subjective report of the participants, this amount of reward manipulation was necessary to perceive the task more difficult or easier at the subjective level. Importantly, this manipulation did not alter the core property of the task, namely, in each pair one option was still better than the other.

### Placebo and nocebo induction and treatment characteristics

In the placebo/nocebo groups, participants were informed that they would receive a combined stimulation of both tDCS and tNILS, however with opposite direction with regards to the declared cognitive effects of the combined intervention. In the placebo group volunteers were provided the information that the intervention would improve their performance by 10 to 25%, whereas in the nocebo-conditioning group participants were informed that the stimulation would decrease their performance by 10 to 25%. The stimulation procedure and the treatment characteristics were identical in both conditioning groups. Participants received two tDCS electrodes bilaterally over the frontal cortices that were positioned according the 10/20 international EEG system. The electrodes (F3 red, anode electrode; F4 blue, cathode electrode; both 3 × 3.5 cm) were fixed by using conductive paste. A formally validated active sham protocol of tDCS was utilized with 20 s fade-in, 30 s stimulation at 1.0 mA and 10 s fade-out period^23^ and impedance was kept below 20 kΩ. Active sham tDCS at 1.0 mA induces cutaneous sensations (e.g., light to moderate amount of itching, tingling or burning) in the overwhelming majority of the participants^22,23^ without inducing aftereffects in the cortical excitability. Moreover, a 3-dimensional (3D) motion analysis system (Zebris Medical GmbH, Germany) was utilized and introduced for the participants as tNILS device. The system consists of a mobile measuring unit of the 3D navigation station and a computer. The mobile measuring unit was additionally fixed with a label ‘Prototype’ written on it and was precisely positioned over the F3 electrode. The operation of the tNILS device was modified in order to produce a constant, subdued buzzing sound and was controlled by one of the primary investigators (Z.T.). The application of the tDCS and the tNILS was synchronized in time. The participants and the operators were all required to close their eyes during stimulation period in order to prevent the laser beam from reaching the eyes and causing possible eye damage. The primary investigator was only present during the preparation of the tNILS and during the one-minute-long stimulation period, during this time he had restricted and standardized communication with the operator and no verbal interaction with the participant. Overall, the operator and the participants were informed that tDCS and tNILS would be concurrently applied for 1 min, despite the fact that only active sham protocol of tDCS was used. The rationale behind employing combined stimulation protocols for placebo and nocebo stimulation was to reduce the possibility that either the operator or the participant encounter contradictory information regarding the efficacy of tDCS on cognitive functions. As remarkably fewer publications are available about tNILS, we minimized the risk that the declared cognitive effects are explicitly questioned. None of the participants or the operator declared any concerns about the stimulation explicitly.

When returned on the second day, participants in the placebo group were informed that their performance improved on the first day. Further, they received the information that on the second day they would perform an even more difficult version of the task. This instruction was necessary, since they performed an instruction-congruent, easier version of the behavioral task (i.e., task 2) on the first day. In order to assess cognitive placebo effect, we used the default reward contingencies in task 3. In the default version (task 3), participants unavoidably encountered less monetary gains than in the manipulated version (task 2). This could have potentially hindered the development of cognitive placebo effect. On the contrary, participants in the nocebo group were informed on the second day that their performance decreased on the previous day and they would perform an easier version of the task. This was necessary, because participants were likely to encounter more monetary gains in task 3 than in the manipulated version (task 2), which may interfere with inducing cognitive nocebo effect. After the written instructions, both groups received the sham NIBS, the procedure of which was identical to the first day. Participants in the placebo and nocebo control groups received no sham NIBS, no instruction either on the first or on the second day.

### Monetary reimbursement

In all groups, partcipants were informed that they would receive 5 EUR/hour as monetary reimbursement for their participation in the study. In addition, they were told that they would receive a performance-dependent monetary bonus after every correct decision. Due to the monetary bonus, participants could increase their base earnings by 70%. At the end of the study, all participants received the same amount of monetary reimbursement independent of their performance (8.5 EUR/hour). The underlying principle of calculating the final amount of earnings remained unknown both to the operator and to the participants. The payment forms were organized by one of the primary investigators (ZT).

### Assessing the expected and the perceived effects in the behavioral performance

On the second day, we assessed explicit expectancy and perceived changes in the behavioral performance by questionnaire. We asked the participants whether they would expect (before learning) or had perceived (after learning) a decline, no impact or improvement in their performance relative to the first day. We asked the following questions: “What do you think, how will you perform today?” to assess expectancies and “What do you think how did you perform today?” to assess experienced performance changes. Note that the participants in the placebo and nocebo groups received verbal suggestions in conjunction with the active sham NIBS protocol while no task manipulation was used on the second day. This allowed us to compare the expected and perceived cognitive performance between task 1 and 3, which mirrors the comparison of the actual cognitive performance between task 1 and 3, aimed to capture the cognitive placebo/nocebo effects.

### Computational Modelling

We modelled individual responses to stimuli using a well-established reinforcement-learning model that was successfully applied to this task in earlier work^18,41^. In this model, an internal value representation *Q* of each possible action *a* is maintained. In our task, each action *a*_*t*_ in trial *t* resulted in an immediate reward *r*_*t*_. Through successive action-reward pairings, the internal reward representation *Q*_*t*_(*a*) for action *a* in trial *t* is updated according to

$${Q}_{t}(a)={Q}_{t-1}(a)+{\alpha }_{G}{[{r}_{t}-{Q}_{t}(a)]}_{+}+{\alpha }_{L}{[{r}_{t}-{Q}_{t}(a)]}_{-}$$

with initial conditions *Q*_0_(*a*) = 0 for all *a*. Here, [*x*]_+_ is equal to *x* whenever *x* ≥ 0 and equal to 0 otherwise. Similarly, [*x*]_−_ is equal to *x* whenever *x* ≤ 0 and 0 otherwise. The parameters *α*_*G*_, *α*_*L*_ ∈ [0, 1] are free parameters that indicate the degree to which the existing value is updated with the prediction error (i.e., they are learning-rates). We specify two separate learning rates for learning from gains and learning from losses because it has been suggested that there are separate underlying neural pathways for reward-based and loss-based learning^41,64^. In addition, in our earlier work, models allowing the learning rates to be different generally allowed for much better model-fits^18^. Here we will use a similar strategy and evaluate model-selection criteria on both a simpler model with a single learning rate and the suggested model that incorporates separate learning rates for gains and losses.

To decide which of two possible actions to take, i.e., choosing stimulus *A* or *B* when both are simultaneously presented, the model compares the relative magnitudes of the current estimates of the value for each action. The probability to choose action *a* is then calculated according to the softmax rule

$${P}_{t}(a)=\frac{\exp (\frac{1}{\beta }{Q}_{t}(a))}{\sum _{a^{\prime} \in \{A,B\}}\,\exp (\frac{1}{\beta }{Q}_{t}(a^{\prime} ))}.$$

The free parameter *β* > 0 specifies the degree to which the agent relies on the value-difference for decision making. The higher this parameter, the more “exploratory” decisions are encouraged, i.e., decisions for the less-valued option become more likely.

For estimating the set of parameters (*α*_*G*,*i*_, *α*_*L*,*i*_, *β*_*i*_) that best reflect the behaviour of subject *i*, we employ a hierarchical Bayesian estimation strategy^65^ that assumes that individual parameter estimates are drawn from a joint group-level distribution. That way, individual estimates are being constrained by each other to avoid unstable estimates that often occur with unconstrained, maximum-likelihood based fits (partial-pooling^44,66^). Specifically, parameters for all subjects for the first session on the first day are constrained to come from group-level distributions

$$\begin{array}{lll}{\rm{probit}}({\alpha }_{G,i}) &  \sim  & {\rm{Normal}}({\mu }_{{\alpha }_{G}},{\sigma }_{{\alpha }_{G}})\\ {\rm{probit}}({\alpha }_{L,i}) &  \sim  & {\rm{Normal}}({\mu }_{{\alpha }_{L}},{\sigma }_{{\alpha }_{L}})\\ \mathrm{log}({\beta }_{i}) &  \sim  & {\rm{Normal}}({\mu }_{\beta },{\sigma }_{\beta }\mathrm{).}\end{array}$$

We also estimated the parameters on the second day for each of the four manipulated groups (group ∈ {placebo, nocebo, placebo control, nocebo control}) with the natural history group (NHG) serving as baseline, by adding a group-specific effect, *b*_*θ*, group_ to the individual parameter estimates for parameter *θ* ∈ {*α*_*G*_, *α*_*L*_, *β*}. Therefore, the day-two estimates for subject *i* who was in group group_*i*_ were estimated as

$$\begin{array}{lll}{\rm{probit}}({\alpha }_{G,i}^{{\rm{day2}}}) &  \sim  & {\rm{Normal}}({\mu }_{{\alpha }_{G}}+{b}_{{\alpha }_{G}}+{b}_{{\alpha }_{G},{{\rm{group}}}_{i}},{\sigma }_{{\alpha }_{G}})\\ {\rm{probit}}({\alpha }_{L,i}^{{\rm{day2}}}) &  \sim  & {\rm{Normal}}({\mu }_{{\alpha }_{L}}+{b}_{{\alpha }_{L}}+{b}_{{\alpha }_{L},{{\rm{group}}}_{i}},{\sigma }_{{\alpha }_{L}})\\ \mathrm{log}({\beta }_{i}^{{\rm{day2}}}) &  \sim  & {\rm{Normal}}({\mu }_{\beta }+{b}_{\beta }+{b}_{\beta ,{{\rm{group}}}_{i}},{\sigma }_{\beta }\mathrm{).}\end{array}$$

In order to stabilize estimates, we constrained the 15 group-level effects *b*_*θ*, group_ by a common distribution

$${b}_{\theta ,{\rm{group}}} \sim {\rm{Normal}}\mathrm{(0},{\sigma }_{b}\mathrm{).}$$

Finally, we chose to use weakly-informative priors that help with constraining complex models to reasonable areas of the parameter space:

$$\begin{array}{lll}{\mu }_{\alpha } &  \sim  & {\rm{Normal}}(\,-\,\mathrm{0.5,}\,\mathrm{0.6)}\\ {\mu }_{\beta } &  \sim  & {\rm{Normal}}(\,-\,\mathrm{1.5,}\,\mathrm{0.8)}\\ {\sigma }_{\alpha } &  \sim  & \mathrm{HalfCauchy}(0\mathrm{.01})\\ {\sigma }_{\beta } &  \sim  & \mathrm{HalfNormal}(0\mathrm{.3})\\ {\sigma }_{b} &  \sim  & {\rm{Normal}}\mathrm{(0,1).}\end{array}$$

**Figure 5:**
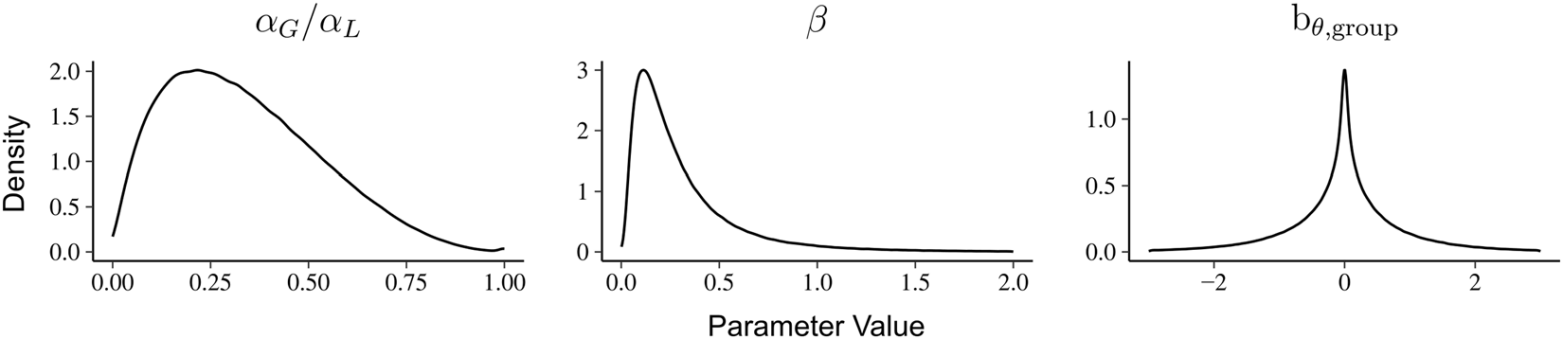
Implied prior on the individual parameter-estimates for the learning rates *α*_*G*_ and *α*_*L*_ and the group-coefficients *b*_*θ*, group_. The prior was chosen to be “weakly informative” such that values that have previously been observed would have a higher probability than uncommon ones. However, the prior still leaves room for more extreme estimates and does not place any group-specific biases.

Here, identical priors were chosen for *α*_*G*_ and *α*_*L*_. The implied prior distributions on the parameters are depicted in Fig. 5. The prior puts more probability on “realistic” estimates of the parameters. For the learning rates, values below 0.5 have a higher probability (consistent with estimates from previous reports^18,41^) while higher values are still permissive. Similarly, for the noise-parameter *β*, lower values are encouraged while high values above 2 are assumed to be unrealistic and are hence discouraged (though not cut off). For reference, a *β* = 2 corresponds to choosing the correct symbol in the easiest symbol-pair (AB), when there is perfect knowledge about the relative values with a probability of only 0.57. Such extreme *β* values would therefore result in almost purely chance performance irrespective of the learning rate. For the group-level coefficients *b*_*θ*, group_, the prior encourages low values (implementing a safeguard against false-positives) but it has long tails towards the extremes, such that strong evidence for the presence of extreme values will be reflected in the posterior.

### Model-fitting procedure

We used Hamiltonian Monte-Carlo (HMC) algorithms implemented in the Stan software^67,68^. The standard Bayesian regression models reported for analysing accuracy, reaction-time and subjectively reported data were fit using the R-package brms^69^ which provides an easy-to-use front-end for Stan. Reinforcement-learning models were fit using eight parallel chains with warm-up period of a 1000 samples each such that 8000 samples were drawn from the converged chains. For the simpler performance model, we used brms’s default of 4 chains with 2000 samples each. Traceplots for all variables were manually screened for convergence. In addition, we calculated the Gelman-Rubin diagnostic^70^ to ensure that all $\hat{R}$ ≤ 1.1. We used the leave-one-out cross-validation information criterion (LOOIC) for model-selection^45^. LOOIC differences larger than 10 can be considered strong^71^.
